# External Ventricular Drain Placement, Critical Care Utilization, Complications, and Clinical Outcomes after Spontaneous Subarachnoid Hemorrhage: A Single-Center Retrospective Cohort Study

**DOI:** 10.3390/jcm13041032

**Published:** 2024-02-11

**Authors:** Abhijit Vijay Lele, Christine T. Fong, Andrew M. Walters, Michael J. Souter

**Affiliations:** Department of Anesthesiology and Pain Medicine, University of Washington, Seattle, WA 98195, USA; cfong@uw.edu (C.T.F.); walters3@uw.edu (A.M.W.); msouter@uw.edu (M.J.S.)

**Keywords:** external ventricular drain, subarachnoid hemorrhage, quality, outcomes, complications, EVD

## Abstract

Background: To examine the association between external ventricular drain (EVD) placement, critical care utilization, complications, and clinical outcomes in hospitalized adults with spontaneous subarachnoid hemorrhage (SAH). Methods: A single-center retrospective study included SAH patients 18 years and older, admitted between 1 January 2014 and 31 December 2022. The exposure variable was EVD. The primary outcomes of interest were (1) early mortality (<72 h), (2) overall mortality, (3) improvement in modified-World Federation of Neurological Surgeons (m-WFNSs) grade between admission and discharge, and (4) discharge to home at the end of the hospital stay. We adjusted for admission m-WFNS grade, age, sex, race/ethnicity, intraventricular hemorrhage, aneurysmal cause of SAH, mechanical ventilation, critical care utilization, and complications within a multivariable analysis. We reported adjusted odds ratios (aORs) and 95% confidence intervals (CI). Results: The study sample included 1346 patients: 18% (n = 243) were between the ages of 18 and 44 years, 48% (n = 645) were between the age of 45–64 years, and 34% (n = 458) were 65 years and older, with other statistics of females (56%, n = 756), m-WFNS I–III (57%, n = 762), m-WFNS IV–V (43%, n = 584), 51% mechanically ventilated, 76% White (n = 680), and 86% English-speaking (n = 1158). Early mortality occurred in 11% (n = 142). Overall mortality was 21% (n = 278), 53% (n = 707) were discharged to their home, and 25% (n = 331) improved their m-WFNS between admission and discharge. Altogether, 54% (n = 731) received EVD placement. After adjusting for covariates, the results of the multivariable analysis demonstrated that EVD placement was associated with reduced early mortality (aOR 0.21 [0.14, 0.33]), an improvement in m-WFNS grade (aOR 2.06 [1.42, 2.99]) but not associated with overall mortality (aOR 0.69 [0.47, 1.00]) or being discharged home at the end of the hospital stay (aOR 1.00 [0.74, 1.36]). EVD was associated with a higher rate of ventilator-associated pneumonia (aOR 2.32 [1.03, 5.23]), delirium (aOR 1.56 [1.05, 2.32]), and a longer ICU (aOR 1.33 [1.29;1.36]) and hospital length of stay (aOR 1.09 [1.07;1.10]). Critical care utilization was also higher in patients with EVD compared to those without. Conclusions: The study suggests that EVD placement in hospitalized adults with spontaneous subarachnoid hemorrhage (SAH) is associated with reduced early mortality and improved neurological recovery, albeit with higher critical care utilization and complications. These findings emphasize the potential clinical benefits of EVD placement in managing SAH. However, further research and prospective studies may be necessary to validate these results and provide a more comprehensive understanding of the factors influencing clinical outcomes in SAH.

## 1. Introduction

Spontaneous subarachnoid hemorrhage (SAH) is a significant global health concern [1]. The estimated global crude incidence for aneurysmal SAH is 6.67 per 100,000 persons, with a wide variation across WHO regions from 0.71 to 12.38 per 100,000 persons. A total of 500,000 individuals are estimated to suffer from aneurysmal SAH each year, with two-thirds in low- and middle-income countries [2]. Its occurrence carries substantial morbidity and mortality. For the clinical care of patients with SAH, placing an external ventricular drain (EVD) represents a timely intervention to treat acute symptomatic hydrocephalus. EVDs are pivotal in mitigating elevated intracranial pressure, facilitating cerebrospinal fluid drainage, and allowing for intracranial pressure and cerebral autoregulation monitoring. Their utility extends across a spectrum of SAH cases, yet the comprehensive evaluation of their effects on clinical outcomes remains an area of ongoing research [3] and clinical interest.

Spontaneous subarachnoid hemorrhage is a common reason for EVD placement in the USA [4]. Published literature on SAH has focused on predicting permanent cerebrospinal fluid drainage, as well as institutional efforts to reduce the rates of EVD-associated infections and hemorrhagic complications. A notable gap persists in our understanding of EVD placement’s broader impact on critical care utilization and complications. This gap in knowledge underscores the compelling need for a more extensive and rigorous exploration of the associations between EVD placement and a spectrum of clinical outcomes.

This study addresses the knowledge gap by examining a cohort of SAH patients. We aimed to illuminate the role of EVD placement in the management of SAH and provide a nuanced understanding of its impacts on critical care utilization and clinical outcomes. Specifically, our investigation explored associations between EVD placement and key outcomes, including early mortality, overall mortality, improvement in neurological status, and the likelihood of being discharged home. We also explore the critical care utilization and the complications observed in patients with EVDs. We employed rigorous adjustments for necessary covariates, such as modified World Federation of Neurosurgical Societies (m-WFNSs) grade [5,6], age, sex, race/ethnicity, language, and mechanical ventilation status. By addressing this knowledge gap, our study sought to offer fresh insights, inform clinical decision-making, and contribute to optimizing care for SAH patients, thereby enhancing their prognosis and overall healthcare outcomes. We tested the hypothesis that clinical outcomes are associated with EVD and that patients with EVD are at higher risk for critical care utilization and complications.

## 2. Materials and Methods

### 2.1. Study Design

This study utilized a retrospective, single-center design to investigate the impacts of external ventricular drain (EVD) placement on critical care utilization, clinical outcomes, critical care utilization, and complications in hospitalized adults with spontaneous subarachnoid hemorrhage (SAH).

### 2.2. Study Population

The study included adult patients aged 18 years and older admitted to Harborview Medical Center, a comprehensive stroke center, between 1 January 2014 and 31 December 2022, with a primary diagnosis of SAH (ICD.9 code 430 (2014–2015)/ICD.10 codes [I60.0-I60.9], (2016–2022)). Patients < 18 years of age and those with SAH secondary to trauma were excluded.

### 2.3. Institutional Practice Regarding EVD Placement and Management after SAH

The decision to place an antimicrobial-impregnated EVD [7] is taken by the attending consultant neurosurgeon. It is typically based on the level of consciousness and presence of hydrocephalus on admission computerized tomogram (CT) of the brain. Before securement of any vascular lesion causing SAH, the EVD is set at +20 cmH20 above the external auditory meatus with a maximal EVD output of 20 mL. After successfully securing the vascular lesion, the EVD is lowered to + 10 cmH20, and patients remain at this setting until the resolution of the highest period of onset of delayed cerebral ischemia, typically occurring by days 10–14. After this, the EVD is raised to +20 cmH20 for 24 h, followed by CT of the brain. If there is no clinical deterioration in the level of consciousness, elevated intracranial pressure, worsening headache, or change in ventricular size on the CT, the EVD is then clamped for 24 h before another repeat CT of the brain; depending upon the CT and examination, a decision is then made to remove the EVD or evaluate for placement of a permanent cerebrospinal fluid diversion device. If intracranial pressure is stable, early mobilization is encouraged based on the bedside nurse or the expert opinion of the physical/occupational therapist evaluation. The EVD is clamped during any out-of-bed mobilization. Patients receive a dose of antibiotics before the placement of the EVD and do not routinely receive antibiotics during the EVD maintenance phase. Cerebrospinal fluid was collected every Monday and Thursday for infectious surveillance.

### 2.4. Data Collection

Patient data were extracted from electronic medical records. The following variables were collected: demographic information: age, sex, race/ethnicity, and language. The collected clinical variables included admission modified World Federation of Neurosurgical Societies (m-WFNS) grade, use of mechanical ventilation, and EVD placement. Primary clinical outcomes were early mortality (<72 h), overall mortality, improvement in w-WFNS grade between admission and discharge, and discharge destination to home.

### 2.5. Primary Outcomes

Our primary outcomes were mortality within the first 72 h of the hospitalization, any mortality during the hospitalization, improvement in the m-WNFS from grade IV–V to grade I–III, and discharge to home. We chose discharge to home as a surrogate for the modified Rankin Scale due to the high predictive values between discharge disposition and modified Rankin Scale scores [8].

### 2.6. Secondary Outcomes

Critical care utilization was categorized as follows: vasopressors in the first 24 h of ICU admission (phenylephrine/norepinephrine), medications to treat intracranial pressure elevation/management of hyponatremia (mannitol, 23.4% saline, 2% saline, 3% saline), neurostimulant for patients with persistent depressed level of consciousness (amantadine), pain, agitation and distress management (oral morphine equivalent use in the ICU, gabapentin, quetiapine, haloperidol, dexmedetomidine), surgical procedures (intracranial/extracranial), invasive monitoring/lines (arterial line/central line), neurophysiological monitoring (automated pupillometer, brief electroencephalography, continuous electroencephalography), other procedures (bronchoscopy for bronchioalveolar lavage, tracheostomy, gastrostomy feeding tube), ancillary services consultation (speech-language pathologist, physical therapy/occupational therapy/rehabilitation medicine), and length of stay (time spent on the mechanical ventilator, intensive care unit length of stay, hospital length of stay). We also report the insertion of a permanent cerebrospinal fluid diversion device and EVD-associated infections.

The complications were categorized as follows: non-infectious (adult respiratory failure, gastrointestinal hemorrhage, ileus, deep venous thrombosis/pulmonary embolism, delirium, days with delirium), and infectious (clostridium. difficile infection, catheter-associated urinary tract infection, ventilator-associated pneumonia, catheter-associated bloodstream infections, and multiple antimicrobial drug resistance).

### 2.7. Statistical Analysis

Descriptive statistics, including means, medians, and frequencies, were calculated to summarize the characteristics of the study population. Categorical variables were presented as proportions and continuous variables as means ± standard deviations or medians with interquartile ranges, as appropriate. Patients were categorized into age categories (18–44, 45–64, and 65 years or older), m-WFNS grade groups (I–III and IV–V), race categories (White/Non-White), and language categories (English/Non-English). We calculated the sample size based on the incidence of outcome variables. Adjustments were made for admission m-WFNS grade, age, sex, race/ethnicity, language, intraventricular hemorrhage, aneurysmal cause of SAH, and mechanical ventilation. Our final sample size of 1346 would provide us a power greater than 80% to examine each of the four outcome measures with a required minimal sample size of 345. Multivariable logistic regression models assessed the associations between EVD placement and clinical outcomes. The Hosmer–Lemeshow goodness-of-fit test was performed to test each model. We presented the multivariable regression results as adjusted odds ratios (aOR) with corresponding 95% confidence intervals (CIs) for the primary outcomes. We considered a *p*-value <0.05 to be statistically significant. Statistical analysis was performed using RStudio (Version: 2023.12.1+402) [9].

## 3. Results

### 3.1. Subsection

#### Study Sample Characteristics

The final study sample comprised patients with a median age of 59 years [IQR 49,68], 44% (n = 590) were males, 57% (n = 762) were with m-WFNS grade I–III, 24% (n = 322) were non-White race/ethnicity, 51% (n = 690) were mechanically ventilated, and 38% (n = 511) were due to aneurysmal etiology.

Table 1 details the study sample characteristics, stratified by the EVD status. After multivariable analysis, we observed that males (aOR 0.71 [0.57, 0.90]) were less likely to undergo EVD placement in multivariable analysis, while mechanically ventilated patients (aOR 2.55 [1.87, 3.47]) were more likely to undergo EVD placement.

### 3.2. Outcomes

#### 3.2.1. Primary Outcomes

Table 2 highlights the primary outcomes in our SAH cohort. Specifically, 20.7% (n = 278) died, 10.5% (n = 142) died within 72 h of admission, 24.6% (n = 331) improved their grade of SAH from m-WNFS IV–V to grade I–III, and 52.5% (n = 707) were discharged home from the hospital. In addition, 14.7% (n = 198) were transitioned to comfort measures only. Notably, 4.2% (n = 57) were declared dead by neurologic criteria, and 8.5% (n = 115) received a permanent cerebrospinal fluid diversion device.

As shown in Table 3, the results of the multivariable analysis note that when adjusted for age, race, language, m-WFNS grade, intraventricular hemorrhage, aneurysmal cause of SAH, and mechanical ventilation, EVD was independently associated with reduced early mortality (aOR 0.21 [0.14, 0.33]) or an improvement in m-WFNS grade (aOR 2.06 [1.42, 2.99]) but not associated with overall mortality (aOR 0.69 [0.47, 1.00]) or being discharged home at the end of the hospital stay (aOR 1.00 [0.74, 1.36]).

#### 3.2.2. Secondary Outcomes

##### Care Utilization by EVD Placement Status

Table 4 details the critical care utilization in SAH patients by their EVD status and adjustment for the m-WNFS category, intraventricular hemorrhage, and aneurysmal cause of SAH.

Table 5 details the critical care utilization in SAH patients by their EVD status and results of the multivariable analysis.

##### Complications

Table 6 details the infectious and non-infectious complications observed in patients by their EVD status using univariable and multivariable analysis. EVD was associated with higher odds of acute respiratory failure (OR 1.78 [1.19, 2.67]), ventilator-associated pneumonia (OR 2.32 [1.03, 5.23]), and delirium (OR 1.56 [1.05, 2.32]).

## 4. Discussion

A retrospective, single-center study investigated critical care utilization, complications, and clinical outcomes in hospitalized adults with spontaneous subarachnoid hemorrhage (SAH) in whom EVDs were placed across admission illness severity. The main findings of this study are that EVD placement is associated with (a) reduced risk of early mortality, (b) improved odds for improvement in m-WFNS, (c) not associated with overall mortality or being discharged to home, (d) increased critical care utilization, and (e) intensive care unit complications.

### 4.1. Impact of EVD Placement on Early Mortality and Improvement in Neurological Recovery

The significant association between EVD placement and reduced early mortality in SAH patients may be attributed to several factors. First, EVDs effectively address elevated intracranial pressure due to acute symptomatic hydrocephalus, a common and life-threatening complication of SAH. EVDs are critical in preventing secondary brain injury during the acute phase of SAH by facilitating cerebrospinal fluid drainage and providing a controlled mechanism for monitoring and regulating intracranial pressure, pain relief, and reduction in nausea. This reduction in intracranial pressure may improve cerebral perfusion, reducing the risk of early mortality. Additionally, EVD placement enables close monitoring and early intervention in cases of deteriorating neurological status, allowing for prompt adjustments in treatment strategies. Even patients with high-grade SAH may show improvements in their level of consciousness after EVD placement. Our results demonstrate that the EVD placement was across m-WNFS grades and confirm our practice that all SAH patients, even those with m-WNFS grades IV and V, should be given a chance to improve their neurological function. Clinicians should not withhold placement of EVD and propagate a self-fulfilling prophecy of poor outcomes until they objectively demonstrate the lack of clinical improvement. Thus, EVD placement can be considered a low-risk and high-reward intervention [3,10]. In patients with acute symptomatic hydrocephalus after SAH, urgent CSF diversion should be performed to improve neurological outcomes as recommended in the 2023 guidelines by the American Heart Association [11]. The neurological status after EVD may determine the “truly resuscitated” clinical grade [12].

### 4.2. Impact of EVD Placement on Discharge Outcomes

The absence of a significant association between EVD placement and the likelihood of being discharged home suggests that a complex interplay of factors beyond EVD placement influences the discharge destination [10]. The patient’s overall health status, functional capacity, and social support networks are likely crucial in determining discharge outcomes. Additionally, post-acute care resources and rehabilitation services may substantially impact the patient’s ability to return home. It is essential to recognize that EVD placement may significantly influence early survival and neurological recovery but may not directly affect the discharge destination factors. Further research is needed to comprehensively explore the multifaceted determinants of SAH patients’ discharge outcomes. In conclusion, the observed associations between EVD placement, improved early survival, and enhanced neurological recovery in SAH patients can be explained by the multifaceted benefits of EVDs in managing intracranial pressure, preventing secondary brain injuries, and enabling timely interventions. However, the complex nature of discharge outcomes underscores the need for a holistic approach to SAH management, considering various patient-specific factors and post-acute care resources.

### 4.3. Impact of EVD on Clinical Care in the Intensive Care Unit

Our study results demonstrate that after adjusting for the m-WNFS grade, EVD placement was associated with higher odds of diagnostic and therapeutic interventions in patients with SAH. The presence of ECVD provides an opportunity for diagnosis of intracranial pressure elevation and affords a therapeutic option by allowing cerebrospinal fluid diversion; thus, the higher rates of mannitol or hypertonic saline use in patients with EVD are not surprising. A higher likelihood of phenylephrine use (which is the most commonly used vasopressor in our ICU) in the first 24 h of admission may imply a complex interplay between intracranial hypertension and cardiac dysfunction after SAH. Pain, agitation, and distress management seem to be a problem in SAH patients with EVD and is the focus of an ongoing randomized clinical trial for headache management using pterygopalatine nerve blocks, ropivacaine, and dexamethasone [13]. The odds for SAH patients undergoing intra- and extracranial procedures are also not surprising, considering that many patients underwent gastrostomy or tracheostomy tube placement and craniotomy or endovascular procedures for diagnostic and therapeutic purposes.

Similarly, arterial line and central line placements, which are not routinely placed in our intensive care unit, emphasize the uniqueness of the SAH patients who undergo EVD placement and the continued complexities of care delivery that may justify their placement. Automated pupillometer, brief and continuous electroencephalography use was higher in SAH patients with EVD, implying their use in coma diagnosis and follow-up. At the time of the study, we did not undertake electroencephalography for early diagnosis of delayed cerebral ischemia, so the results suggest that the use of electroencephalography was more to rule out convulsive/non-convulsive status epilepticus in patients with continued depressed levels of consciousness.

### 4.4. Impact of EVD on Complications

Patients undergoing EVD placement face an elevated risk of both infectious and non-infectious complications, as evidenced by the data analysis. These complications can significantly impact patient outcomes and hospital resources. A multifaceted approach is essential in addressing these quality improvement (QI) opportunities. Non-infectious complications, such as acute respiratory failure and delirium, were more prevalent among patients receiving EVD when adjusted for covariates. Additionally, these patients often require prolonged hospitalization, prolonged mechanical ventilation, and urinary catheterization, all of which contribute to the increased risk of infections. To reduce their occurrence, healthcare providers should prioritize early mobilization, vigilant monitoring, and aggressive management of these complications. This may include strategies to optimize respiratory function, proactive interventions to prevent ileus, and tailored approaches to delirium prevention and management. Concurrently, EVD patients also face an increased risk of infectious complications, including ventilator-associated pneumonia. To mitigate these infections, stringent infection control practices must be upheld. This includes a focus on oral hygiene, elevation of the head of the bed, and careful maintenance of ventilator-associated equipment, which is crucial to prevent VAP. Regular data collection surveillance and feedback mechanisms can facilitate continuous improvement efforts. By analyzing infection rates and non-infectious complications associated with EVD placement, healthcare facilities can identify trends and areas for intervention. This data-driven approach enables targeted strategies to reduce the occurrence of these complications, enhance patient safety, and optimize resource allocation.

In summary, the analysis underscores the importance of QI initiatives in reducing complications related to EVD placement. By addressing both infectious and non-infectious complications through a comprehensive approach that combines clinical vigilance, infection control, and data-driven interventions, healthcare providers can enhance patient care and outcomes in this vulnerable population. It is reassuring to note that our cohort’s overall rate of EVD-associated meningitis was low at 1.2%, emphasizing the importance of using an EVD insertion and maintenance bundle. To put into perspective, cumulatively, our EVD cohort had an average of over 275 h of monitoring; thus, the rates are lower than previously reported.

### 4.5. Study Implications, Clinical Relevance, and Areas for Quality Improvement

Based on data from our study, EVD placement is associated with a significant increase in diagnostic and therapeutic intensity in patients with SAH. The length of stay in our study is similar to that published by Nelson et al. [3]. Hospital length of stay improvement should be closely evaluated based on the advantages of rapid EVD weaning [14,15], since that is not our current institutional practice. Early mobilization is found to be safe and feasible [16,17]. In our study, patients with EVD had a higher likelihood of obtaining physical/occupational therapist and rehabilitation expert consultation, implying that these patients may need ongoing support to meet their ambulatory needs. Our current routine practice encourages early mobility, discussed during daily morning rounds attended by the bedside nurse, emphasizing the importance of building and implementing a shared mental model. EVD placement should not be considered an absolute contraindication to mobility; however, clinically, mobility typically occurs after securing the underlying cause of SAH and ensuring clinical stability, based on the level of consciousness, while balancing the risk for falls. In addition, EVDs are frequently clamped while moving/transporting. Thus, information regarding EVD clamp tolerance is equally essential to ensure safe intrahospital transport [18]. Lastly, in our study, not all high-grade m-WFNS patients received an EVD, which is an opportunity for quality improvement.

Considering our study’s findings, it becomes evident that EVD placement in SAH patients is akin to a necessary but essential intervention. While it may introduce clinical care complexities in the short term, its positive impact on patient outcomes cannot be overstated. The observed complexities in clinical care should not be viewed solely as adverse outcomes but as aspects of SAH management requiring vigilant monitoring and proactive management. Healthcare providers must be prepared to address these challenges promptly, implementing evidence-based interventions to minimize complications associated with EVDs. As clinicians, our responsibility lies in recognizing the potential challenges posed by EVD placement and addressing them proactively. This entails vigilant monitoring, evidence-based management strategies, and interdisciplinary collaboration to optimize patient care. The findings of this study underscore the delicate balance between clinical care complexities and the benefits of EVD placement in the context of SAH management.

### 4.6. Limitations

This retrospective study offers valuable insights into the relationship between external ventricular drain (EVD) placement, critical care utilization, complications, and clinical outcomes in hospitalized adults with spontaneous subarachnoid hemorrhage (SAH). However, it is important to acknowledge several limitations. The retrospective design relies on historical data, potentially subject to inaccuracies and documentation issues. Unmeasured confounding variables, alternative models focusing on subgroup analyses, temporal changes in medical practices, and potential selection bias among SAH patients who received EVD were not fully addressed. Furthermore, the study’s single-center nature and a relatively higher rate of non-aneurysmal SAH may limit the generalizability of the findings, and statistical considerations, including alternative modeling approaches, were not explored. These limitations should be considered when interpreting and applying the study’s results to clinical practice. Future research with prospective designs and broader data collection may help mitigate some of these limitations.

## 5. Conclusions

In conclusion, our study sheds light on the interplay between EVD placement and clinical outcomes in SAH patients. It highlights the indispensable role of EVDs in reducing mortality and enhancing neurological recovery, even in the face of associated clinical care complexities. These complexities should not deter clinicians but serve as a call for diligent and informed management. EVD placement, while at times a necessary challenge, ultimately contributes to improved patient outcomes and underscores the resilience of modern neurocritical care in the face of complex clinical scenarios.

## Figures and Tables

**Table 1:** Characteristics of patients with spontaneous subarachnoid hemorrhage admitted between 2014 and 2022 stratified by placement of an external ventricular drain.

Characteristic	Overall N = 1346	No EVD N = 615	EVD N = 731	Univariable OR [95%CI]	Multivariable aOR [95% CI]
Age in years	59 (49, 68)	58 (48, 68)	60 (50, 68)	NA	NA
Age category					
Age 18–44 years	243 (18%)	123 (20%)	120 (16%)	Reference	Reference
Age 45–64 years	645 (48%)	287 (47%)	358 (49%)	1.28 [0.95;1.72]	1.26 [0.92, 1.72]
Age ≥ 65 years	458 (34%)	205 (33%)	253 (35%)	1.26 [0.93;1.73]	1.18 [0.85, 1.65]
Sex					
Female	756 (56%)	317 (52%)	439 (60%)	Reference	Reference
Male	590 (44%)	298 (48%)	292 (40%)	0.71 [0.57;0.88]	0.71 [0.57, 0.90]
m-WFNS grade					
I–III	762 (57%)	423 (69%)	339 (46%)	Reference	Reference
IV–V	584 (43%)	192 (31%)	392 (54%)	2.54 [2.04;3.19]	1.32 [0.96, 1.82]
Intraventricular hemorrhage	504 (37.4%)	127(20.7%)	377 (51.6%)	4.09 [3.21;5.22]	5.12 [3.83;6.84]
Race category					
White	1024 (76%)	480 (78%)	544 (74%)	Reference	Reference
Non-White	322 (24%)	135 (22%)	187 (26%)	0.82 [0.63;1.05]	0.89 [0.68, 1.18]
Language category					
English	1,158 (86%)	540 (88%)	618 (85%)	Reference	Reference
Non-English	188 (14%)	75 (12%)	113 (15%)	1.32 [0.96;1.81]	1.10 [0.78, 1.55]
Mechanical ventilation	680 (51%)	218 (35%)	462 (63%)	3.12 [2.50;3.91]	2.55 [1.87, 3.47]
Co-morbidities					
Diabetes	153 (11.4%)	79 (12.8%)	74 (10.1%)	0.76 [0.54;1.07]	0.61 [0.41, 0.92]
Chronic hypertension	704 (52.3%)	316 (51.4%)	388 (53.1%)	1.07 [0.86;1.33]	0.52 [0.40, 0.69]
Aneurysmal cause of SAH	511 (38.0%)	191 (31.1%)	320 (43.8%)	1.73 [1.38;2.17]	1.67 [1.28, 2.17]
Transition to comfort measures only	198 (14.7%)	66 (10.7%)	132 (18.1%)	1.83 [1.34;2.53]	1.12 [0.77, 1.64]

Abbreviations: EVD: external ventricular drain; m-WFNS: modified World Federation of Neurosurgeons SAH grade; OR: unadjusted odds ratio; aOR; adjusted odds ratio.

**Table 2:** Associations between external ventricular drain placement and clinical outcomes.

Characteristic	Overall N = 1346	No EVD N = 615	EVD N = 731
Overall mortality	278 (20.7%)	168 (23.0%)	110 (17.9%)
Early mortality (mortality < 72 h of admission)	142 (10.5%)	83 (13.5%)	59 (8.1%)
Improvement in m-WFNS grade between admission and discharge	331 (24.6%)	89 (14.5%)	242 (33.1%)
Discharge to home from the hospital	707 (52.5%)	379 (61.6%)	328 (44.9%)

Abbreviations: EVD: external ventricular drain; m-WFNS: modified World Federation of Neurosurgeons SAH grade.

**Table 3:** Clinical outcomes in patients with spontaneous subarachnoid hemorrhage by their external ventricular drain placement status.

	Early Mortality	Overall Mortality	Improvement in m-WFNS Grade	Discharged Home
Model Intercept	0.01 [0.00, 0.02]	0.01 [0.01, 0.02]	0.00 [0.00, Inf]	11.63 [7.11, 19.03]
EVD placement	0.25 [0.16, 0.39] *	0.69 [0.47, 1.00]	1.66 [1.12, 2.45] *	1.00 [0.74, 1.36]
Hosmer–Lemeshow goodness-of-fit test *p*-Value	0.2332	0.7913	0.7293	0.1567

Notes: Model adjusted for age, sex, Race, Language, m-WFNS grade, intraventricular hemorrhage, aneurysmal cause of SAH, and mechanical ventilation. * Indicates Bonferroni-corrected *p*-Values < 0.05

**Table 4:** The Associations between external ventricular drain placement and critical care utilizations after spontaneous subarachnoid hemorrhage n.

	No EVD	EVD	Odds Ratio [95% CI]	Adjusted Odds Ratio [95% CI]
	N = 615	N = 731		
Vasopressors within 24 h of admission				
Phenylephrine	15 (2.44%)	53 (7.25%)	3.10 [1.77;5.77]	2.40 [1.30, 4.43]
Norepinephrine	57 (9.27%)	91 (12.4%)	1.39 [0.98;1.98]	0.87 [0.58, 1.29]
Medications to treat intracranial pressure elevation/management of hyponatremia				
Mannitol	32 (5.20%)	94 (12.9%)	2.68 [1.78;4.12]	1.77 [1.13, 2.78]
23.4% saline	5 (0.81%)	32 (4.38%)	5.43 [2.29;16.2]	5.59 [2.16, 14.42]
2% saline	23 (3.74%)	99 (13.5%)	4.01 [2.55;6.55]	4.03 [2.53, 6.43]
3% saline	25 (4.07%)	75 (10.3%)	2.69 [1.71;4.37]	2.70 [1.69, 4.30]
Amantadine for a persistent depressed level of consciousness	14 (2.28%)	38 (5.20%)	5.43 [2.29;16.2]	1.63 [0.84, 3.18]
Pain, agitation, and distress management				
Gabapentin	160 (26%)	267 (36.5%)	1.64 [1.29;2.07]	1.86 [1.43, 2.41]
Quetiapine	49 (7.97%)	172 (23.5%)	3.54 [2.54;5.02]	3.16 [2.21, 4.52]
Haloperidol	36 (5.85%)	90 (12.3%)	2.25 [1.52;3.41]	2.14 [1.39, 3.28]
Dexmedetomidine	27 (4.39%)	101 (13.8%)	3.47 [2.27;5.49]	2.52 [1.58, 4.03]
Surgical procedures				
Intracranial	125 (20.3%)	325 (44.5%)	3.13 [2.46;4.01]	4.03 [3.02, 5.39]
Extracranial	116 (18.9%)	253 (34.6%)	2.27 [1.77;2.94]	2.44 [1.88, 3.16]
Invasive monitoring				
Arterial line	28 (4.55%)	94 (12.9%)	3.08 [2.02;4.85]	2.32 [1.46, 3.69]
Central line	23 (3.74%)	118 (16.1%)	4.92 [3.16;8.00]	3.90 [2.41, 6.32]
Neurophysiological monitoring				
Automated pupillometer	145 (23.6%)	381 (52.1%)	3.52 [2.79;4.47]	2.94 [2.22, 3.90]
Brief electroencephalography	68 (11.1%)	85 (11.6%)	1.06 [0.75;1.49]	0.91 [0.63, 1.31]
Continuous electroencephalography	56 (9.11%)	140 (19.2%)	2.36 [1.70;3.31]	1.94 [1.36, 2.77]
Other procedures				
Bronchoscopy for bronchioalveolar lavage	5 (0.81%)	35 (4.79%)	5.97 [2.53;17.7]	4.68 [1.76, 12.41]
Tracheostomy	7 (1.14%)	33 (4.51%)	4.03 [1.87;10.1]	3.60 [1.54, 8.42]
Gastrostomy feeding tube	28 (4.55%)	78 (10.7%)	2.49 [1.61;3.96]	1.79 [1.11, 2.88]
Ancillary services consultation				
Speech-language pathologist	438 (71.2%)	609 (83.3%)	2.02 [1.55;2.62]	1.80 [1.34, 2.40]
Physical therapy	470 (76.4%)	614 (84.0%)	1.62 [1.23;2.13]	1.48 [1.09, 2.00]
Occupational therapy	463 (75.3%)	609 (83.3%)	1.64 [1.25;2.14]	1.46 [1.08, 1.97]
Rehabilitation medicine	23 (3.74%)	73 (9.99%)	2.84 [1.78;4.70]	3.78 [2.28, 6.27]

**Table 5:** The associations between external ventricular drain placement and pain, agitation and distress management and length of stay.

	No EVD	EVD	Adjusted Odds Ratio [95% CI]	*p*-Value
	N = 615	N = 731		
Pain, agitation, and distress management				
Oral morphine equivalent use in the ICU *	195 (427)	580 (1682)	1.00 [1.00;1.00]	0.0004
Length of stay				
Time spent on the mechanical ventilator #	0.97 (4.17)	3.97 (9.23)	1.15 [1.11;1.20]	<0.0001
Intensive care unit length of stay **	4.97 (4.22)	13.9 (7.64)	1.33 [1.29;1.36]	<0.0001
Hospital length of stay **	10.1 (12.0)	22.2 (18.2)	1.09 [1.07;1.10]	<0.0001

* Model adjustment for the m-WNFS category, presence of intraventricular hemorrhage, aneurysmal cause of SAH, craniotomy procedure, and mechanical ventilation. ** Model adjustment for the m-WNFS category, presence of intraventricular hemorrhage, aneurysmal cause of SAH, craniotomy procedure, and mechanical ventilation. # Model adjustment for the m-WNFS category, presence of intraventricular hemorrhage, aneurysmal cause of SAH, and craniotomy procedure.

**Table 6:** The association between external ventricular drain placement and infectious and non-infectious complications.

	No EVD	EVD	Unadjusted Odds Ratio [95%CI]	Adjusted Odds Ratio [95% CI]
	N = 615	N = 731		
Non-infectious complications				
Acute respiratory failure	43 (6.99%)	112 (15.3%)	2.40 [1.67;3.51]	1.78 [1.19, 2.67]
Gastrointestinal hemorrhage	6 (0.98%)	3 (0.41%)	0.43 [0.09;1.69]	0.29 [0.07, 1.27]
Ileus	22 (3.58%)	45 (6.16%)	1.76 [1.06;3.02]	1.44 [0.83, 2.52]
Deep venous thrombosis/pulmonary embolism	17 (2.76%)	30 (4.10%)	1.50 [0.83;2.81]	1.18 [0.62, 2.26]
Delirium	87 (14.1%)	308 (42.1%)	4.64 [3.51;6.19]	1.56 [1.05, 2.32]
Days with delirium	6.89 (19.7)	15.0 (23.7)	1.02 [1.01;1.02]	
Infectious complications				
EVD-associated infection	NA	17 (1.2%)	NA	NA
Clostridium. difficile infection	3 (0.49%)	10 (1.37%)	2.73 [0.82;12.8]	2.69 [0.70, 10.30]
Catheter-associated urinary tract infection	7 (1.14%)	28 (3.83%)	3.40 [1.55;8.59]	2.19 [0.90, 5.29]
Ventilator-associated pneumonia	8 (1.30%)	38 (5.20%)	4.09 [1.99;9.58]	2.32 [1.03, 5.23]
Catheter-associated bloodstream infections	0	0	NA	
Multiple antimicrobial drug resistance	0	0	NA	

Model adjusted for m-WFNS category, intraventricular hemorrhage, aneurysmal cause of SAH, and mechanical ventilation.

## Data Availability

Data is unavailable due to privacy restrictions.
